# Effects of traditional Chinese medicine combined with modern rehabilitation therapies on motor function in children with cerebral palsy: A systematic review and meta-analysis

**DOI:** 10.3389/fnins.2023.1097477

**Published:** 2023-02-08

**Authors:** Zhengquan Chen, Zefan Huang, Xin Li, Weiwei Deng, Miao Gao, Mengdie Jin, Xuan Zhou, Qing Du

**Affiliations:** ^1^Department of Rehabilitation, Xinhua Hospital, School of Medicine, Shanghai Jiao Tong University, Shanghai, China; ^2^Chongming Hospital, Shanghai University of Medicine and Health Sciences, Shanghai, China

**Keywords:** cerebral palsy, traditional Chinese medicine, acupuncture, motor function, systematic review

## Abstract

**Objective:**

Traditional Chinese Medicine (TCM) has considerable experience in the treatment of cerebral palsy (CP), but little evidence shows the effect of a combination of TCM and modern rehabilitation therapies on CP. This systematic review aims to evaluate the effect of integrated TCM and modern rehabilitation therapies on motor development in children with CP.

**Methods:**

We systematically searched five databases up to June 2022, including PubMed, the Cumulative Index to Nursing and Allied Health, Cochrane Library, Embase, and Web of Science. Gross motor function measure (GMFM) and Peabody Development Motor Scales-II were the primary outcomes to evaluate motor development. Secondary outcomes included the joint range of motion, the Modified Ashworth scale (MAS), the Berg balance scale, and Activities of Daily living (ADL). Weighted mean differences (WMD) and 95% confidence intervals (CIs) were used to determine intergroup differences.

**Results:**

A total of 2,211 participants from 22 trials were enrolled in this study. Among these, one study was at a low risk of bias and seven studies showed a high risk of bias. Significant improvements were found in GMFM-66 (WMD 9.33; 95% CI 0.14–18.52, *P* < 0.05, *I*^2^ = 92.1%), GMFM-88 (WMD 8.24; 95% CI 3.25–13.24, *P* < 0.01, *I*^2^ = 0.0%), Berg balance scale (WMD 4.42; 95% CI 1.21–7.63, *P* < 0.01, *I*^2^ = 96.7%), and ADL (WMD 3.78; 95% CI 2.12–5.43, *P* < 0.01, *I*^2^ = 58.8%). No adverse events were reported during the TCM intervention in the included studies. The quality of evidence was high to low.

**Conclusion:**

Integrated TCM and modern rehabilitation therapies may be an effective and safe intervention protocol to improve gross motor function, muscle tone, and the functional independence of children with CP. However, our results should be interpreted carefully because of the heterogeneity between the included studies.

**Systematic review registration:**

https://www.crd.york.ac.uk/PROSPERO/, identifier CRD42022345470.

## 1. Introduction

Cerebral palsy (CP) is a group of persistent motor and postural development disorders with participation limitations caused by non-progressive brain lesions (Michael-Asalu et al., 2019). CP is mainly divided into seven types: spastic quadriplegia, spastic diplegia, spastic hemiplegia, dyskinesia, ataxia, Worster-Drought syndrome, and mixed types (Chinese Association of Rehabilitation Medicine Pediatric Rehabilitation Committee et al., 2022a). Most children with CP are spastic (85–91%), with symptoms of muscle stiffness (Novak et al., 2017).

Cerebral palsy is a frequent cause of physical disability in children. Functional independence and social participation may be influenced by motor dysfunction, pain, and cognitive impairment in children with CP (Lindsay, 2016). Because of the irreversible brain damage in CP, comprehensive rehabilitation therapies for motor function, cognition, language, and daily living ability are widely used in children with CP (Vargus-Adams and Martin, 2011). Evidence showed that exercise interventions significantly improved gait speed and muscle strength in children with CP (Liang et al., 2021). Invasive therapies, such as selective dorsal rhizotomy, botulinum toxin-A therapy, and intrathecal baclofen therapy, were effective in reducing muscle tone, which can be employed in children with spastic CP (Damiano et al., 2021).

Traditional Chinese medicine (TCM) has been reported as an alternative therapy to CP treatment. CP is described as “congenital deficiencies,” “retardation,” and “weakness” in the view of TCM. Congenital deficiencies lead to loss of nutrients in the meridians and musculoskeletal system, which prevents the meridians from mobilizing the bones, causing joint stiffness. The spleen and stomach promote the development of the children *via* nutrient absorption, and weakness of the spleen and stomach are associated with a deficiency of kidney essence, which results in developmental delay. The treatment strategy is to stretch and dredge the meridian, and warm and nourish the spleen and kidney to condition the poor state of the congenial deficiencies and the acquired weakness of the spleen and kidney. At the same time, treatment should also focus on mind refreshing and wisdom increasing, because of the lesions of the brain in children with CP. TCM treatments, which include massage, acupuncture, herbs, and Qigong, have been practiced in the treatment of CP for a long time. One study showed that TCM may improve the posture balance and cognition of rats with CP (Niu et al., 2021). Increased secretion of dopamine, brain-derived neurotrophic factor, and nerve growth factor were discovered after stimulations on the meridian in rats, which helped repair neuronal damage (Chuang et al., 2007; Tao et al., 2016). Studies suggested that massage and acupuncture dilate blood vessels and increase blood flow and oxygen supply by stimulating acupoints in children with CP, which may improve the function and metabolism of brain cells and muscles (Wang and Wu, 2005; Wu et al., 2008).

Although modern rehabilitation therapies were widely recommended by the guidelines as the first-line treatment for children with CP (Castelli and Fazzi, 2016; Verschuren et al., 2016; Damiano et al., 2021), there was a paucity of evidence on whether TCM is a beneficial supplement to modern rehabilitation therapy to improve the motor development in children with CP. This systematic review aims to clarify the effectiveness of integrated TCM and modern rehabilitation therapies on motor development compared to modern rehabilitation therapies only in children with CP, and we hypothesize that the combination of TCM and modern rehabilitation therapies may be better in the improvement of motor development than modern rehabilitation therapies only.

## 2. Materials and methods

This systematic review was conducted under the guidance of the Cochrane Handbook for Systematic Reviews of Interventions (Cumpston et al., 2019). To find relevant studies published until June 2022, we systematically searched PubMed, the Cumulative Index to Nursing and Allied Health, Cochrane Library, Embase, and Web of Science. Search terms, such as “traditional Chinese medicine,” “TCM,” “cerebral palsy,” and “motor function,” were used, and the full search strategy was listed in the Supplementary material. The references of enrolled studies were screened to find additional eligible articles. The protocol of this systematic review has been registered in PROSPERO (No. CRD42022345470).

### 2.1. Eligibility criteria

The enrolled studies should meet the following conditions: (1) Participants: infants or children under 18 years old with a clear diagnosis of CP and with abnormalities in motor function or postural development. (2) Interventions: TCM treatments combine with conventional modern rehabilitation therapies. (3) Comparisons: conventional modern rehabilitation like physical therapy, occupational therapy, or speech therapy. (4) Outcome measures: primary outcome measures: ➀ Gross motor function measure (GMFM)-66 and GMFM-88 (Te Velde and Morgan, 2022): measurement of motor function of decubitus position, turn-over, sitting position, creeping, and kneeling, erect position, walking, running, and jumping. ➁ Fine motor function measure: 2 subscales (grasping and visual-motor integration) of Peabody Development Motor Scales-II (Fay et al., 2019). Secondary outcome measures: ➀ Muscle tone: Joint range of motion and modified Ashworth scale (MAS) (Bohannon and Smith, 1987). ➁ Balance function: the Berg balance scale (Downs, 2015). ➂ Activities of daily living (ADL): cerebral palsy specified ADL scale to measure functional independence (Deng et al., 2005; Zhang and Hu, 2012). (5) Study design: Randomized Controlled Trial (RCT). (6) Language: English and Chinese.

### 2.2. Exclusion criteria

(1) Patients with serious heart, lung, or nerve diseases. (2) Concomitant invasive treatments in the intervention group or the control group, including surgery and botulinum toxin injecting. (3) Any TCM treatment that was used in control groups.

### 2.3. Data extraction

Two reviewers (Z.C. and Z.H.) independently screened the enrolled articles through title and abstract screening and full-text reading. Data were extracted under the guidance of Cochrane Collaboration by Z.C. and Z.H. Extracted data included: publication year, author name, demographics, the severity of CP, data on outcome measures, and adverse events. A third reviewer (Q.D.) would participate in the discussion when disagreements arise.

### 2.4. Quality assessment

The Cochrane risk of bias tool was used to assess the methodological quality of the included studies. The evidence quality was measured by Grading of Recommendation Assessment, Development, and Evaluation (GRADE). The quality appraisal was done by Z.H.

### 2.5. Statistical analysis

Stata version 16.0 (StataCorp, College Station, TX, USA) was used for data analysis. The fixed effects model would be chosen for quantitative analysis if there was no significant heterogeneity, otherwise random effects model would be used. The results would be presented as weighted mean difference (WMD) and 95% confidence interval (CI) with a significance value set as 0.05. Data would be synthesized if there were over two TCM intervention groups in one research. Heterogeneity was determined by I^2^ statistics with a significant value set as 50%. A sensitivity test was used to identify the outlying studies that may influence the between-study heterogeneity. Review Manager 5.0 (The Cochrane Collaboration, Copenhagen, Denmark) was used to generate the bias chart of risk of bias evaluation.

## 3. Results

**FIGURE 1:**
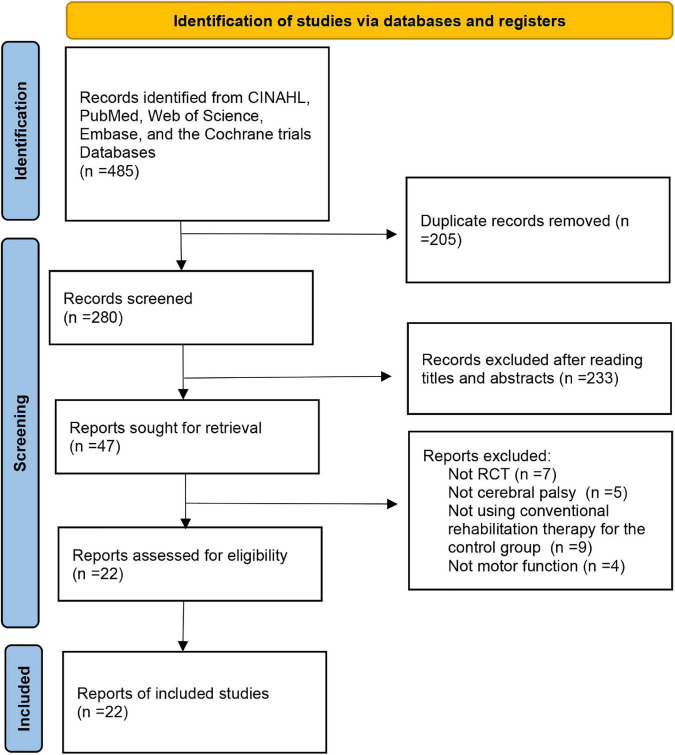
Flowchart of the process of literature search and extraction of studies meeting the inclusion criteria.

We obtained 485 relevant papers from the five databases, and 205 articles were removed as duplicates. The remind 280 articles were evaluated based on the relevance and publication type, and 233 articles with obvious irrelevant topics or non-RCTs were ruled out. After the full-text reading, 22 RCTs inclusion met the eligibility criteria. The screening flow diagram is described in Figure 1.

### 3.1. Studies’ characteristics

The demographics of the included studies, consisting of author year, number of participants, age, type of CP, outcome measures, significant results, and loss to follow-up, are shown in a customized Table 1. The intervention method, treatment frequency, and treatment duration of the intervention group and the control group are shown in Table 2.

**TABLE 1:** Baseline demographic and clinical characteristics of study participants.

	References	Study type	No. of participants (% girls)	Age: range/mean (SD)	Severity and classification of CP	TCM intervention	Outcome measures	Adverse events	Time points	Dropout rate
1	Dabbous et al., 2016 #	randomized controlled Trial	T: 40 (42.5) I: 20 C: 20	T: 36 (2) mo.	Hemiplegic spastic CP	Low-level laser on body acupuncture points	1. MAS 2. Wrist and ankle range of motion 3. GMFM-88	Not provided	I: Baseline 3 months C: Baseline 3 months	I: 0% C:0% T:0%
2	Deng et al., 2005 #	Randomized controlled trial	T: 90 (33.3) I: 60 (33.3) C: 30 (33.3)	T: 34 between 1–3 y. 56 between 4–7 y.	Severity: T: 47 mild, 22 moderate, 21 severe I: 32 mild, 15 moderate, 13 severe C:15 mild, 7 moderate, 8 severe Classification: T: 44 spastic, 22 dyskinetic, 4 hypotonia, 20 mixed I: 30 spastic, 15 dyskinetic, 2 hypotonia, 13 mixed C: 14 spastic, 7 dyskinetic, 2 hypotonia, 7 mixed	Scalp acupuncture	1. Motor function 2. ADL 3. Social adaptation DQ	Not provided	I: Baseline 2 months C: Baseline 2 months	I: 0% C: 0% T: 0%
3	Duncan et al., 2012	Randomized controlled trial	T: 75 (40) I: 46 (37) C: 29 (55)	I: 28.2 (12.2) months. C: 29.6 (14.9) months.	GMFCS: I: 14 Level I 15 Level II 16 Level III C: 13 Level I 6 Level II 10 Level III Classification: T: spastic CP	Scalp and body acupuncture	1. GMFM-66 2. PEDI-FS	Not provided	I: Baseline 12 weeks 4 weeks gap 12 weeks follow-up C: Baseline 12 weeks 4 weeks 12 weeks acupuncture	I: 8% C: 29.3% T: 17.6%
4	Ji et al., 2019 #	Randomized controlled trial	T: 220 (38.2) I: 110 (37.3) C: 110 (39.1)	I: 3.6 (0.9) year. C: 3.7 (0.8) year.	GMFCS: I: 75 Level I&II 35 Level III C: 72 Level I&II 38 Level III Classification: Spastic CP I: 77 quadriplegia, 33 diplegia C: 72 quadriplegia, 38 diplegia	Body acupuncture	1. GMFM-88 2. FMFM 3. Comprehensive functional score 4. Brain Doppler ultrasound	Not provided	I: Baseline 3 months C: Baseline 3 months	I: 0% C: 0% T: 0%
5	Ji et al., 2008	Randomized controlled trial	T: 80 (50) I: 40 (47.5) C: 40 (52.5)	I: 5.15 (2.78) year. C: 6.04 (2.37) year.	Spastic CP	Scalp acupuncture	1. GMFM 2. Functional Independence Measure	Not provided	I: Baseline 3 months C: Baseline 3 months	I: 0% C: 0% T: 0%
6	Li J. et al., 2021 #	Randomized controlled trial	T: 28 (32.1) I: 14 (35.7) C: 14 (28.6)	I: 64.42 (21.10) months. C: 69.28 (18.15) months.	GMFCS: I: 8 Level I 3 Level II 3 Level III C: 5 Level I 6 Level II 3 Level III Classification: Spastic CP I: 5 hemiplegia, 9 diplegia C: 6 hemiplegia, 8 diplegia	Wrist-ankle acupuncture	1. GMFM-66 2. Modified Tardieu Scale 3. Motor Evoked Potentials	Not provided	I: Baseline 4 weeks C: Baseline 4 weeks	I: 14.3% C: 7.1% T: 10.7%
7	Li et al., 2017 #	Randomized controlled trial	T: 300 (32.3) I: 150 (35.3) C: 150 (29.3)	I: 3.5 (1.44) year. C: 3.58 (0.88) year.	Classification: I: 116 spastic, 2 ataxic, 9 mixed, 23 dyskinetic C: 115 spastic, 15 mixed, 20 dyskinetic	Scalp acupuncture	1. GMFM-88 2. Gesell scale 3. Head MRI/CT	Not provided	I: about 6 months C: about 6 months	T: 0
8	Liu et al., 2013#	Randomized controlled trial	T: 200 (24) I: 100 (31) C: 100 (17)	I: 66 between 1–3 years. 34 between 3–7 years. C: 72 between 1–3 years. 28 between 3–7 years.	Classification: I: 78 spastic, 4 ataxic, 10 dyskinetic, 23 hypotonia C: 71 spastic, 14 dyskinetic, 13 hypotonia, 2 ataxic	Body acupuncture	1. GMFM-66 2. DQ of gross motor, fine motor, social adaptation 3. Head MRI/CT	None	I: Baseline 90 days 1 year follow up C: Baseline 90 day 1 year follow up	I: 0% C: 8% T: 4%
9	Luo et al., 2020 #	Randomized controlled trial	T: 60 (43.3) I: 30 (46.7) C: 30 (40)	I: 2.7 (0.6) year. C: 2.5 (0.7) year.	Spastic CP	Scalp acupuncture	1. GMFM-88 D and E 2. Berg balance scale	Not provided	I: Baseline 6 months C: Baseline 6 months	T: 0
10	Mo et al., 2016	Randomized controlled trial	T: 70 (44.3) I: 35 C: 35	T: 30 (10) months.	Spastic CP	TCM herb fumigation	1. FMFM scale	Not provided	I: Baseline 3 months C: Baseline 3 months	T: 0
11	Qi and Wang, 2018 #	Randomized controlled trial	T: 120 (36.7) I: 80 (37.8) C: 40 (32.5)	I: 29.5 (15.0) months. C: 27 (8) months.	GMFCS: I: 52 Level III 28 Level IV C:21 Level III 19 Level IV Classification: I: 53 spastic, 3 ataxic, 13 dyskinetic, 3 hypotonia, 8 mixed C: 28 spastic, 5 dyskinetic, 3 hypotonia, 4 mixed	Body acupuncture	1. Surface electromyography 2. GMFM-88 3. Berg balance scale	Not provided	I: Baseline 12 weeks C: Baseline 12 weeks	I: 7.5% C: 7.5% T: 7.5%
12	Shen et al., 2017 #	Randomized controlled trial	T: 60 (46.7) I: 30 (40) C: 30 (53.3)	I: 202.3 (6.4) days C: 196.4 (5.6) days	Spastic CP	Body Acupuncture +TCM tuina	1. GMFM -88 dimension ABCD 2. Muscle tone of gastrocnemius muscle	Not provided	I: Baseline 6 months C: Baseline 6 months	T: 0
13	Wang et al., 2011 #	Randomized controlled trial	T: 120 (36.7) I: 60 (38.3) C: 60 (35)	I: 3.26 (2.05) year. C: 3.51 (1.83) year.	Spastic CP Severity: I: 7 mild, 39 moderate, 14 severe C: 8 mild, 39 moderate, 13 severe	Body acupuncture	1. MAS 2. GMFM-88 3. Comprehensive Function Assessment	None	I: Baseline 3 months C: Baseline 3 months	T:0
14	Wang et al., 2008 #	Randomized controlled trial	T: 60 (40) I: 30 (43.3) C: 30 (36.7)	I: 6–36 months. C: 6–36 months.	Spastic CP I: 25 diplegia, 4 hemiplegia, 1 triplegia. C: 24 diplegia, 4 hemiplegia, 2 triplegia.	TCM tuina	1. MAS 2. GMFM-66	Not provided	I: Baseline 3 months C: Baseline 3 months	T:0
15	Zhang and Du, 2013 #	Randomized controlled trial	T: 120 (47.5) I: 60 (48.3) C: 60 (46.7)	I: 13.6 (4.2) months. C: 13.9 (4.9) months.	Classification: I: 32 spastic, 2 ataxic, 9 dyskinetic, 16 hypotonia, 1 mixed C: 34 spastic, 2 ataxic, 10 dyskinetic, 14 hypotonia	Electric acupuncture	GMFM -88 dimension B	Not provided	I: Baseline 4 weeks C: Baseline 4 weeks	T:0
16	Zhang and Liu, 2018 #	Randomized controlled trial	T: 90 I: 60 C: 30	I: 3.76 (0.88) months. C: 3.72 (0.89) months.	Spastic CP	Body acupuncture	1. Clinical spasm index 2. MAS 3. Surface electromyogram	Not provided	I: Baseline 20 days C: Baseline 20 days	T: 0
17	Zhang et al., 2020 #	Randomized controlled trial	T: 118 (39.8) I: 79 (39.2) C: 39 (41.0)	I: 38 (2) months. C: 37 (2) months.	Spastic CP	Scalp acupuncture	1. MAS 2. Wrist active range of motion 3. Grasping and Visual-motor integration of Peabody Developmental motor scale-II	Not provided	I: Baseline 6 months C: Baseline 6 months	I: 1.67% C: 2.5% T: 1.25%
18	Zhang N. et al., 2014	Randomized controlled trial	T: 80 (41.3) I: 40 (37.5) C: 40 (45)	I: 4 (1) year. C: 4 (1) year.	Spastic CP	Scalp acupuncture	1. GMFM-88 dimensions D and E 2. MAS	Not provided	I: Baseline About 75 days C: Baseline About 75 days	T:0
19	Zhang et al., 2007	Randomized controlled trial	T: 40 I: 21 C: 19	I: 21.75 (10–60) months. C: 23.63 (4–60) months.	I: 3 spastic hemiplegia, 14 spastic diplegia, 2 ataxic, 1 hypotonic, 1 quadriplegia C: 2 spastic hemiplegia, 9 spastic diplegia, 3 ataxic, 1 quadriplegia, 3 hypotonic, 1 mixed	Body acupuncture	1. GMFM-88 2. Comprehensive function	Not provided	I: Baseline 6 months 12 months C: Baseline 6 months 12 months	T:0
20	Zhang N. X. et al., 2014	Randomized Controlled trial	T: 60 (35) I: 30 (50) C: 30 (20)	I: 28.7 (13.8) months. C: 34.9 (15.0) months.	Not provided	Body acupuncture	1. GMFM-66	Not provided	I: Baseline 6 months 12 months C: Baseline 6 months 12 months	T:0
21	Zhang and Hu, 2012 #	Randomized controlled Trial	T: 60 (41.7) I: 30 (46.7) C: 30 (36.7)	I: 4.3 (2–12) years. C: 4.1 (2–13) years.	31 spastic, 2 dyskinetic, 8 ataxic, 12 hypotonia, 7 mixed	Scalp acupuncture	1. Berg balance scale 2. ADL	Not provided	I: Baseline Intervention for 28 days or until hospital discharge C: Baseline Intervention for 28 days or until hospital discharge	T:0
22	Zhao et al., 2017 #	Randomized controlled trial	T: 120 (38.3) I: 60 (36.7) C: 60 (40)	I: 2.9 (1.0) year. C: 2.8 (1.0) year.	Spastic CP	Body acupuncture	1. GMFM-88 2. ADL 3. Grasping and Visual-motor integration of Peabody Developmental motor scale-II	Not provided	I: Baseline 120 days C: Baseline 120 days	T:0

T, total participants; I, intervention group; C, control group; GMFM, gross motor function measure; CP, cerebral palsy; GMFCS, the Gross motor function classification system; ADL, activities of daily life living; DQ, developmental quotient; MAS, Modified Ashworth scale; FMFM, fine motor function measure; PEDI-FS, pediatric evaluation of disability inventory-functional skills; ^#^Included in meta-analysis.

**TABLE 2:** Traditional Chinese medicine combined with rehabilitation and control interventions in the included trials.

	References	Traditional Chinese medicine combined with rehabilitation in the intervention group	Control group intervention	Duration
1	Dabbous et al., 2016 #	Laser acupuncture: Yanglingquan (GB 340), Hegu (LI 4), Zhouliao (LI 12), Taichong (Liv 3) Low-level laser 650 nm, 50 mW power, each point 30 s, energy density: 1.8 J/cm^2^; 2 days/week. + Conventional physiotherapy Same as the control group.	Physiotherapy	3 months
2	Deng et al., 2005 #	Protocol 1: Scalp acupuncture: Foot motor sensory area, Motor Area, The Second Speech Area, and the Third Speech Area, Intellectual area. Needles retained for 1 h, performed 3 times, 300 times/min, 1 time/day, 6 days/week. Protocol 2: Scalp acupuncture+ modern rehabilitation Same as the control group.	Rehabilitation training therapy: Head control training, upper and lower limb exercise training, correct posture training, turning over, from supine to sitting position, and standing training. OT: Functional OT, training in ADL, and controlling eating, dressing and undressing, and urination.	10 weeks
3	Duncan et al., 2012	Acupuncture: Scalp+ body; Manual+ electrostimulation. 5 times/week. +Massage 5 times/week. + Conventional therapies (PT/OT/HT) Same as the control group.	Conventional therapies: PT: gross motor tasks: rolling, sitting, transitions, independent sitting, walking, and stair climbing/ OT: fine motor tasks: eye-hand coordination and ADL/ HT: relaxation in warm water.	12 weeks
4	Ji et al., 2019 #	Acupuncture: Zusanli (ST36), Xuanzhong (GB39), Sanyinjiao (SP6), Pishu (BL20), Shenshu (BL23), Qihai (CV6), Quchi (LI11), Neiguan (PC6), Hegu (LI4), Tianshu (ST25) + Rehabilitation training Same as the control group. + Transcranial magnetic stimulation Same as the control group.	Rehabilitation training: Bobath, Assistive device training, reflex inhibition patterns, key points of control, hand function training 30 min/time, 1 time/day, 5 days/week. Transcranial magnetic stimulation: 1 Hz, 20 min/time, 1 time/day, 5 days/week.	3 months
5	Ji et al., 2008	Scalp acupuncture: Both sides of the motor area, balance area, sensory area, tremor control area, foot motor sensory area, the second speech area and the third Speech area, Baihui (GV 20) and Sishencong (EX-HN 1)° Retained for 30 s, performed every 15 min, 100 times/min, 45 min/time, 1 times/day, 5 days/week. + Exercise therapy Same as the control group.	Exercise therapy: Bobath, recumbent position, rolling over, sitting position, crawling, kneeling and standing position, walking. 45 min/time, 2 times/day, 5 days/week.	3 months
6	Li J. et al., 2021 #	Wrist-ankle acupuncture: Upper 4, upper 5, lower 1, and lower 4 on the affected side. 30 min/time, 1 time/day, 5 days/week. +Routine rehabilitation+ 5-Hz rTMS Same as the control group.	Routine rehabilitation: Stretch and strength training. + Hz rTMS 15 min, 40 stimulation+1,000 pulses/time, 5 days/week.	4 weeks
7	Li et al., 2017 #	Governor Vessel-unblocking and brain-refreshing Scalp acupuncture: Shenting (GV 24), Qianding (GV 21), Houding (GV 19), Toulinqi (GB 15), Touwei (ST 8), Sishencong (EX-HN 1), Motor Area, Foot Motor-sensory Area, and Three Brain Needles on both sides. Adjunct points: The Second Speech Area and the Third Speech Area. Retain 1–3 h, lift and twist 3 times, 1–3 min/time, more than 200 r/min. +Electricstimulation Shenting (GV 24), Houding (GV 19) and Motor Area WQ1002k Han’s treatment apparatus, 2 Hz to 15–100 Hz, 2.5 s/time, 15 min/time, 3 times/week. + Rehabilitation therapies Same as the control group.	Rehabilitation therapies: PT (Bobath method, Ueda method, and Vojta method), structured teaching, ST, and music therapy. 1–2 h/time, 1 time/day.	About 6 months
8	Liu et al., 2013 #	Acupuncture therapy: Clearing the Governor Vessel needling: Yaoshu (GV 2), Yaoyangguan (GV 3), Mingmen (GV 4), Xuanshu (GV 5), Jizhong (GV 6), Zhongshu (GV 7), Jinsuo (GV 8), Zhiyang (GV 9), Lingtai (GV 10), Shendao (GV 11), Shenzhu (GV 12), Taodao (GV 13) and Dazhui (GV 14), Shenshu (BL 23), Taixi (KI 3), Yanglingquan (GB 34), Zusanli (ST 36), and Sanyinjiao (SP 6). Refreshing the mind needling: Shenting (GV 24) to Qianding (GV 21), Qianding (GV 21) to Baihui (GV 20), Baihui (GV 20) to Naohu (GV 17), and Sishencong (Ex-HN 1). Scalp motor area, scalp foot motor sensory area and balance area, Speech areas 1, 2, and 3. Retain 4 h/time and twirling 3 times for 1–3 min, 200 times/minute, every 2 days/time, 10 days/month. + Rehabilitation training Same as the control group.	Rehabilitation training: PT (Bobath therapy) / OT / ST 1–2 h/day, 7 days/week.	3 months
9	Luo et al., 2020 #	Scalp acupuncture: The motor area, foot motor sensory area, balance area, and parietal temporal anterior oblique line. Twist 200 times/minute for 2 min. Retain 1 h/time, manipulate every 30 min, 1 time/day, 5 days/week. + Rehabilitation training Same as the control group.	Rehabilitation training: Practice training, balance training, spasmotherapy apparatus, electromyography biofeedback apparatus, and orthotics. 5 days/week, Orthotics: at least 4 h/day.	6 months
10	Mo et al., 2016	Chinese herbal fumigation: 19 grams of *Eucommia ulmoides* (Chuanduzhong), 19 grams of *Chaenomeles speciosa* (Sweet) Nakai (Mugua), 12 grams of *Angelica sinensis* (Danggui), 16 grams of *Heracleum hemsleyanum* Diels (Duhuo), 6 grams of Cinnamon (Guipi), 18 grams of Chuanduan, 10 grams of Fangfeng, 14 grams of *Ramulus mori* (Sangzhi), 12 grams of *Ramulus cinnamomi* (Guizhi), 12 grams of *Lycopodium japonicum* Thunb (Shenjincao), 10 grams of *Acanthopanax senticosus* (Wujiapi), 10 grams of Mori folium (Sangye), 12 grams of Radix Paeoniae Rubra (Chishao), 16 grams of *Taxillus sutchuenensis* (Lecomte) Danser (Sangjisheng), 10 grams of *Astragalus* (Huangqi). Temperature: 38°C∼40°C, 20 min/time 1 time/day, 5 days/week. + OT Same as the control group.	Routine OT: Passive activity, Bobath, hand support training, weight-bearing training on the affected side, separate movement of arm and shoulder girdle, correct abnormal shoulder posture, grasping ability under visual guidance, restriction-induced training for children with hemiplegia, both hands coordination training, ADL training.	3 months
11	Qi and Wang, 2018 #	Protocol 1: Intradermal needling: Lumbar Yange Gate (DU3), Mingmen (DU4), L2∼L5 Jiaji (EX-B2) Retain 24 h, compress 1 min/time, 80–120 times/minute, moderate force, pause more than 4 h between two compressions, 3 times/day, 5 days/week. + Rehabilitation training Same as the control group. Protocol 2: Acupuncture Lumbar Yange Gate (DU3), Mingmen (DU4), L2∼L5 Jiaji (EX-B2) 30 min/time, 1 times/day, 5 days/week. + Rehabilitation training Same as the control group.	Rehabilitation training: Head-up training, rollover training, sitting training, crawling training, kneeling training, standing walking training, passive movement, active movement, static balance training, dynamic balance training, postural control training, support training, and Bobath. 30 min/time, 1 times/day, 5 days/week.	3 months
12	Shen et al., 2017 #	Acupuncture: Acupoints on the head: Baihui (GV 20), Sishencong (EX-HN 1), Zhisanzhen [Shenting (GV 24), bilateral, Benshen (GB 13)], Niesanzhen. Other acupoints: Shenshu (BL 23), Mingmen (GV 4), Ganshu (BL 18), Pishu (BL 20), Huantiao (GB 30), Weizhong (BL 40), Chengshan (BL 57), Kunlun (BL 60), Futu (ST 32), Zusanli (ST 36), Jiexi (ST 41) and Sanyinjiao (SP 6). every other day. + Tuina: First step: An-pressing, Na-grasping, plucking method, and dot-pressing method to relax spasm; Second step: Digital An-pressing, Kou-knocking, and Gun-rolling method; Apply digital An-pressing, Kou-knocking, and Gun-rolling manipulations to the muscles of the disadvantaged side of the spasm; Third step: Bashen-pulling and Yao shaking to the hip joint, knee joint, and ankle joint; Fourth step: Nie-pinching spine manipulation, An-pressed and Rou-kneaded Ganshu (BL 18), Pishu (BL 20), and Shenshu (BL 23). every other day. + Rehabilitation treatment Same as the control group.	Rehabilitation treatment: Bobath. 2 times/day, 30 min/time.	6 months
13	Wang et al., 2011 #	Acupuncture: Bladder meridian Yuzhen (BL 9) and Tianzhu point (BL 10) connection. Twist: 1 min, retain: 30 min, 1 time/day, 7 days/week. + Rehabilitation training Same as the control group.	Rehabilitation training: Exercise therapy: Bobath and Vojta 45 min/time. OT 30 min/time. ST 30 min/time. 1 time/day, 7 days/week.	3 months
14	Wang et al., 2008 #	Tuina with manipulation of SMKT: Massaging of spine Governor Vessel (DU), Bladder Meridian of Foot-Taiyang (BL), and symptomatic massage for head and limbs. 5 days/week. + Rehabilitation training Same as the control group.	Rehabilitation training: Bobath 1–2 time/day, 30 min/time.	3 months
15	Zhang and Du, 2013 #	EA: Apply to Mingmen (GV4), Jizheng (GV6), Shenshu (BL23), and Pishu (BL20) 4 Hz, tolerable strength; 1 time/day, 30 min/time, 5 days/week. + Sitting training Same as the control group. + Conventional exercise therapy + Hyperbaric Oxygen Therapy	Sitting training: Assist-sitting, legs-crossing-sitting, sitting with one-leg extending, long-term sitting, balancing-sitting, chair-climbing, and prone hand-supporting. 2 time/day, 15–20 min/time, 5 days/week. + Conventional exercise therapy + Hyperbaric Oxygen Therapy	4 weeks
16	Zhang and Liu, 2018 #	Protocol 1: yin-meridian group: Xuehai (SP 10), Yinlingquan (SP 9), Sanyinjiao (SP 6), Taixi (KI 3), and Taichong (LR 3) along yin meridians. Retain 15 min (no retain for frail children), once each other day, 9 a.m.-12 p.m. + Routine rehabilitation treatment Same as the control group. Protocol 2: yang-meridian group: Futu (ST 32), Zusanli (ST 36), Yanglingquan (GB 34), Guangming (GB 37) and Xuanzhong (GB 39) along yang meridians Retain: 15 min (no retain for frail children), once each other day, 9 a.m.-12 p.m. + Routine rehabilitation treatment Same as the control group.	Routine rehabilitation treatment: Exercise therapy (Bobath and Vojta), OT, ST, music psychotherapy, and cognitive rehabilitation. each other day, 40 min/time.	20 days
17	Zhang et al., 2020 #	Protocol 1: Jin’s three-needle therapy Nie sanzhhen, Zhisanzhen, Naosanzhen, Sishenzhen, Dingshenzhen, Shousanzhen, and Shouzhizhen Scalp acupuncture: 1 h/time; Body acupuncture: 30 min/time 1 time/day, 5 days/week. + Conventional OT Same as the control group. Protocol 2: Conventional OT +MyoTrac biostimulation Therapy Same as the control group. + Jin’s three-needle therapy	Conventional OT: Bobath, affected limb muscle strength training, restriction-induced exercise therapy, bimanual coordination training, and ADL. 1 time/day, 30 min/time, 5 days/week. +MyoTrac biostimulation therapy: Placed electrodes at the origin and insertion point of the extensor carpi radialis muscle of the affected limb. EMG-Stim mode, Part 1 (upper limb), “Auto Threshold Adjustment Mode,” “Low Arm Strength,” and perform active wrist dorsiflexion and relaxation according to the “work-rest” prompt. 1 time/day, 15 min/time, 5 days/week.	6 months
18	Zhang N. et al., 2014	Jin three-needle therapy: Sishenzhen, Naosan needle, Zhisanzhen, Temporal (Nie) three-needle, Zusanzhen, Xisanzhen, Chengjin (BL56), Chengshan (BL57). Retain:30 min (If the patient does not cooperate, use rapid twisting, each point 1 min), 1 time/day. + MOTOmed Same as the control group. + Conventional rehabilitation training Same as the control group.	MOTOmed: Connect with the competitive players in “Fitness e-Road Ride” to play bicycle competitive games; 1. Resistance: 0∼10 N.m, 2∼3 min passive training, speed: 5∼30 r/min. 2. Video simulation competition: 6–7 min, active training. 3. Passive relaxation training: 2–3 min. 4. Active training: 6–7 min. 1 time/day, 20 min/time. + Conventional rehabilitation training: 1. Flex the hip, flex the knee, dorsiflexion, and bridge-like exercise; 2. Hip abduction, extend the knee and ankle dorsiflexion, bridge exercise with both knees extended; 3. Abduction and external rotation of both hip joints, alternately extending and flexing both lower limbs are performed repeatedly; 4. The therapist supports the children’s knee and foot and induces dorsiflexion of the ankle. Repeat each movement 5 times; 1 time/day, 30 min/time.	20 Times as a course of treatment, 3–5 days rest between courses, 3 courses of treatment
19	Zhang et al., 2007	Acupuncture: Body Acupuncture: Bai hui (GV 20), Zusanli (ST 36), Quchi (LI 11), Huantiao (GB 30), Yinlingquan (GB 34), Yanglingquan (GB 34), Sanyinjiao (SP 6), Qiangjian (GV18), Yamen (GV15), Fengchi (GB20), Hegu (LI4) Xuanzhong (GB 39). No retain. Scalp acupuncture: Zhisanzhen, Naosanzhen, Balance Zone, Motor Zone. Retain for 1 h, no twisting, every other day. + Rehabilitation training Same as the control group.	Rehabilitation training: PT: (Bobath and Vojta): turn over, abdominal crawling, four-point hold, kneeling position, standing balance training, position conversion between lying and sitting positions, weight loss walking training, calf triceps stretch, single-leg weight-bearing on the right lower extremity, etc. 45 min/time, 5 days/week. OT: (upper extremity fine motor and ADL): cognitive improvement, upper extremity fine motor training, midline hand-eye coordination, two-hand coordination movement training, and two-hand synergy training. 30 min/time, 5 days/week. ST: (promoting language development level and dysarthria training): gesture-symbol stage training, language imitation training. 30 min/time, 5 days/week.	6 months
20	Zhang N. X. et al., 2014	Acupuncture treatment: Body acupuncture: Jiaji (EX-B2), Jianyu (LI 15), Qiichi (LI 11), Hegii (LI 4), Yanglingquan (GB 34), Yinlingquan (SP 9), Xuanzhong (GB 39), Zusanli (ST 36), Sanylnjiao (SP 6), Chengshan (BL 57), TaichOng (LR 3), Taixi (KI 3), Shenmen (HT 4), and heat-reinforcing manipulation. Twist at Jiaji (EX-B2) and pull it out immediately. 7 days/week. Scalp acupuncture: Baihui GV 20), Sishencong (EX-HN 1), Zhisanzhen, Naosanzhen, Niesanzhen and motor area. Retained 1 h without manipulation, every other day. + Rehabilitation training Same as the control group.	Rehabilitation training: Bobath and PDMS-2 exercise training. 5 days/week, 40 min/time.	6 months
21	Zhang and Hu, 2012 #	Scalp acupuncture: Parietal region: Between Baihui (GV 20) and Qiandfng (GV 21), and four parallel lines. Sub-occipital region: Two lines between Naohu (GV 17) to Fengfii (GV 16), Yuzhen (BL 9) to Tianzhu (BL 10). Twist 200 times/min, retain 8 h/time, twist every 30 min for two times, then twist every 2 h; once a day. + Rehabilitation training Same as the control group.	General rehabilitation therapy: Bobath and Vojta. Training raise head, turning over, creeping, sitting, kneeling, standing with a ladder chair, moving with an assistant, standing and walking by oneself. Playing games and recreation. 7 days/week, 40 min/time. + Balance training: General balance training: (Bobath therapy), Provide an unbalanced location, letting children return to the neutral or balanced place by themself. Visual feedback: posture mirror. Decrease or increase muscle tonus. Correcting abnormally developed muscles and bones. 7 days/week, 40 min/time.	3 months
22	Zhao et al., 2017 #	Acupuncture: Baihui (DU20), Fengfu (GV16), Shenzhu (GV12), Zhiyang (GV9), Jinsuo (GV8), Yaoyangguan (GV3), Mingmen (GV4), Pishu (BL20), Shenshu (BL23), Zusanli (ST 36), Sanyinjiao (SP 6). Retain 10 min/time, once every other day. + Physiotherapeutic and hand function training Same as the control group.	Physiotherapeutic and hand function training: Bobath: 40 min/time. hand function training: 20 min/time. 7 days/week.	Acupuncture: 10 times of treatment as a course of treatment Control Group: 20 days of treatment as a course of treatment. The interval between courses of treatment is 20 days, a total of 3 courses of treatment.

^#^Included in meta-analysis. OT, occupational therapy; ADL, activities of daily living; PT, physical therapy; HT, hydrotherapy; GMFM, gross motor function measure; ST, speech therapy; TMS, transcranial magnetic stimulation; SMKT, supplementing marrow and kneading tendon; EA, electroacupuncture; PDMS-2, peabody developmental motor scales 2nd edition.

This review includes 22 articles published from 2005 to 2021, and data from 15 studies were included in the meta-analysis. This systematic review included 2,211 children aged 4 months to 13 years old. The participants in both the intervention group and control group were diagnosed with spasticity, dyskinesia, ataxia, or mixed types of CP depending on the types of motor abnormalities. Hemiplegia, diplegia, or quadriplegia is further diagnosed according to the affected body parts in children with spasticity CP (Sellier et al., 2016; Novak et al., 2017). All included studies were RCTs in English and Chinese language.

The duration of intervention for children with CP ranged from 20 days to 6 months, and the frequency of treatment ranged from 2 to 7 days per week. The duration of TCM treatments was 10–240 min with a median of 45 min. The duration of modern rehabilitation therapies ranged from 30 min to 240 min, and the median duration was 55 min. All participants received conventional modern rehabilitation therapy, and the intervention group was additionally treated with TCM. Modern rehabilitation therapy includes physiotherapy (Zhang et al., 2007; Duncan et al., 2012; Liu et al., 2013; Li et al., 2017), occupational therapy (Zhang et al., 2007; Wang et al., 2011; Duncan et al., 2012; Liu et al., 2013; Zhang and Liu, 2018), speech therapy (Zhang et al., 2007; Wang et al., 2011; Liu et al., 2013; Li et al., 2017; Zhang and Liu, 2018), and hydrotherapy (Dabbous et al., 2016). Fifteen studies explicitly used Bobath therapy (Zhang et al., 2007, 2020; Ji et al., 2008, 2019; Wang et al., 2008, 2011; Zhang and Hu, 2012; Liu et al., 2013; Zhang N. X. et al., 2014; Mo et al., 2016; Li et al., 2017; Shen et al., 2017; Zhao et al., 2017; Qi and Wang, 2018; Zhang and Liu, 2018), and 5 conducted ADL training (Deng et al., 2005; Zhang et al., 2007, 2020; Duncan et al., 2012; Mo et al., 2016). Zhang et al. (2020) used biofeedback therapy, and Zhang N. et al. (2014) added virtual reality games for treatment. In the TCM treatment of the intervention group, 21 studies applied acupuncture treatment, 2 studies used massage therapy (Wang et al., 2008; Shen et al., 2017), and 1 study applied TCM fumigation (Mo et al., 2016). Particularly, 10 studies applied scalp acupuncture (Deng et al., 2005; Zhang et al., 2007, 2020; Ji et al., 2008; Duncan et al., 2012; Zhang and Hu, 2012; Liu et al., 2013; Zhang N. X. et al., 2014; Li et al., 2017; Luo et al., 2020).

### 3.2. Primary outcomes

#### 3.2.1. Gross motor function measure

**FIGURE 2:**
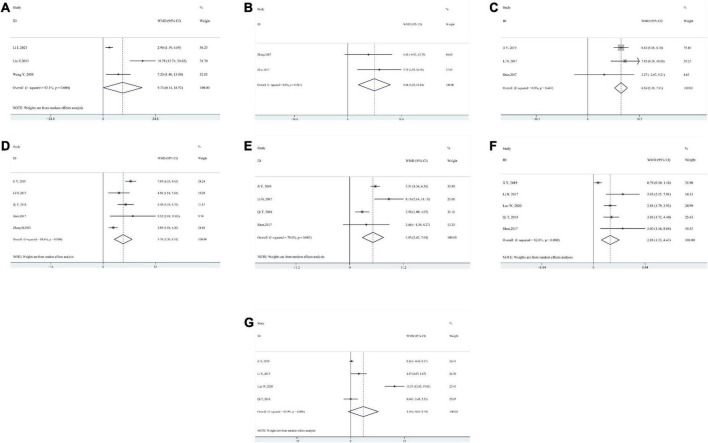
Forest plot of pooled results for gross motor function measure (GMFM) **(A)** GMFM–66. **(B)** GMFM–88. **(C)** GMFM–88–Dimension A. **(D)** GMFM–88–Dimension B. **(E)** GMFM–88–Dimension C. **(F)** GMFM–88–Dimension D. **(G)** GMFM–88–Dimension E.

Four studies evaluated gross motor function according to GMFM-66 (Wang et al., 2008; Duncan et al., 2012; Liu et al., 2013; Li J. et al., 2021). One study provided average GMFM-66 scores without standard deviation, which was excluded from the meta-analysis (Duncan et al., 2012). The pooled analysis reported a significantly better improvement in GMFM-66 in intervention groups than in control groups (WMD 9.33; 95% CI 0.14–18.52, *P* = 0.047, *I*^2^ = 92.1%) (Figure 2A). When Liu et al. (2013) was excluded from the pooled results according to the sensitivity analysis, there were still significant differences between the two groups without significant heterogeneity (WMD 3.30; 95% CI 1.62–4.97, *P* < 0.001, *I*^2^ = 47.4%).

GMFM-88 consists of 88 items in five dimensions: dimension A (lying and rolling), dimension B (sitting), dimension C (crawling and kneeling), dimension D (standing), and dimension E (walking, running, and jumping). Twelve studies applied GMFM-88 or part of GMFM-88 to measure gross motor function. We analyzed each of the five dimensions and the total score of GMFM-88.

The sum of five dimension scores of GMFM-88: Pooled data from 2 studies (Zhang et al., 2007; Zhao et al., 2017) showed a significant result (WMD 8.24; 95% CI 3.25–13.24, *P* = 0.001, *I*^2^ = 0.0%) (Figure 2B).

Dimension A: Pooled data from 3 studies (Li et al., 2017; Shen et al., 2017; Ji et al., 2019) showed a significant result (WMD 6.63; 95% CI 5.36–7.91, *P* < 0.001, *I*^2^ = 0.0%) (Figure 2C).

Dimension B: Pooled data from 5 studies (Zhang and Du, 2013; Li et al., 2017; Shen et al., 2017; Qi and Wang, 2018; Ji et al., 2019) showed a significant result (WMD 5.76; 95% CI 3.20–8.31, *P* < 0.001, *I*^2^ = 84.6%) (Figure 2D). After removing Zhang and Du (2013) based on sensitivity analysis, the pooled results still showed significant differences between the intervention group and the control group while there was no significant heterogeneity (WMD 7.07; 95% CI 5.90–8.23, *P* < 0.001, *I*^2^ = 27.4%).

Dimension C: Pooled data from 4 studies (Li et al., 2017; Shen et al., 2017; Qi and Wang, 2018; Ji et al., 2019) showed a significant result (WMD 4.83; 95% CI 2.62–7.04, *P* < 0.001, *I*^2^ = 79.6%) (Figure 2E). After removing Qi and Wang (2018), there were still significant differences between the intervention group and the control group without significant heterogeneity (WMD 5.49; 95% CI 4.60–6.38, *P* < 0.001, *I*^2^ = 47.7%).

Dimension D: Pooled data from 5 studies (Li et al., 2017; Shen et al., 2017; Qi and Wang, 2018; Ji et al., 2019; Luo et al., 2020) showed a significant result (WMD 2.88; 95% CI 1.33–4.43, *P* < 0.001, *I*^2^ = 82.6%) (Figure 2F). After removing Ji et al. (2019) from the pooled data based on sensitivity analysis, there was no significant statistical heterogeneity, but the intervention group still showed a significantly better effect than the control group (WMD 3.18; 95% CI 2.39–3.97, *P* < 0.001, *I*^2^ = 0.0%).

Dimension E: Pooled data from 4 studies (Li et al., 2017; Qi and Wang, 2018; Ji et al., 2019; Luo et al., 2020) showed no significant result (WMD 4.49; 95% CI −0.82 to 9.79, *P* = 0.097, *I*^2^ = 95.9%) (Figure 2G). After removing Luo et al. (2020) from the pooled results based on the sensitivity analysis, no significant results were found (WMD 0.39; 95% CI −0.27 to 1.05, *P* = 0.248, *I*^2^ = 37.7%).

#### 3.2.2. Fine motor function measure

**FIGURE 3:**
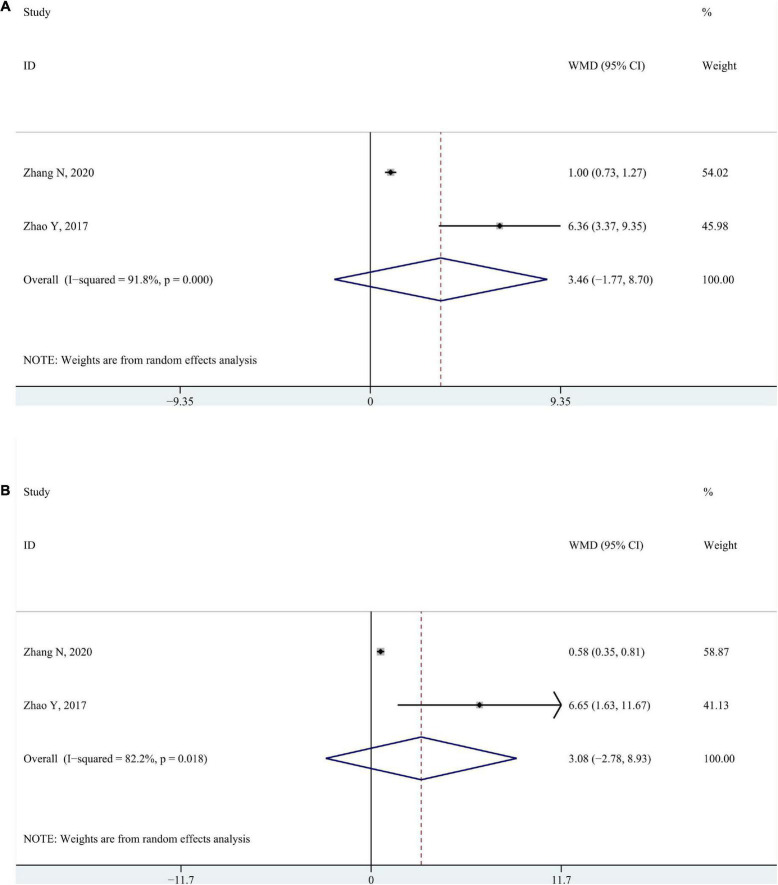
Forest plot of pooled results for Peabody Development Motor Scales-II **(A)** Peabody Developmental Motor Scales–Grasping. **(B)** Peabody Developmental Motor Scales–Visual motor integration.

Two studies, respectively measured fine motor development using the grasping and visual-motor subscales of the Peabody Development Motor Scales-II (Zhao et al., 2017; Zhang et al., 2020). No significant changes were found in neither grasping part (WMD 3.46; 95% CI −1.77 to 8.70, *P* = 0.195, *I*^2^ = 91.8%) nor visual-motor integration part (WMD 3.08; 95% CI −2.78 to 8.93, *P* = 0.303, *I*^2^ = 82.2%) (Figures 3A, B).

### 3.3. Secondary outcomes

#### 3.3.1. Joint range of motion

**FIGURE 4:**
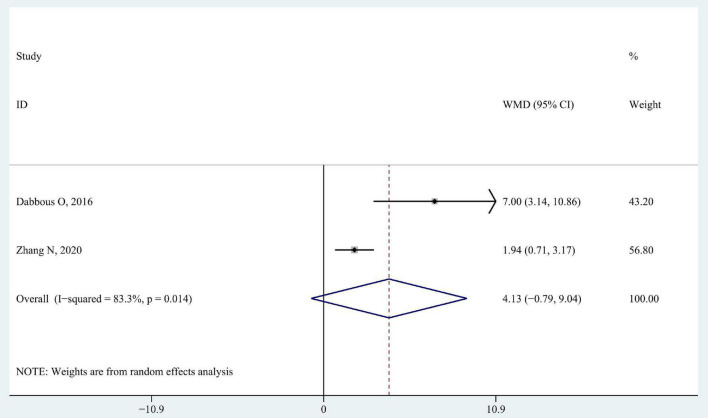
Forest plot of pooled results for joint range of motion.

Two studies that recruited children with hemiplegia spastic CP used the joint range of motion to measure the changes in spasticity. Dabbous et al. (2016) described flexion, and extension of the wrist and ankle while Zhang et al. (2020) measured the wrist active extension. It showed no significant differences in wrist extension range of motion when data were pooled (WMD 4.13; 95% CI −0.79 to 9.04, *P* = 0.100, *I*^2^ = 83.3%) (Figure 4).

#### 3.3.2. Modified Ashworth scale

**FIGURE 5:**
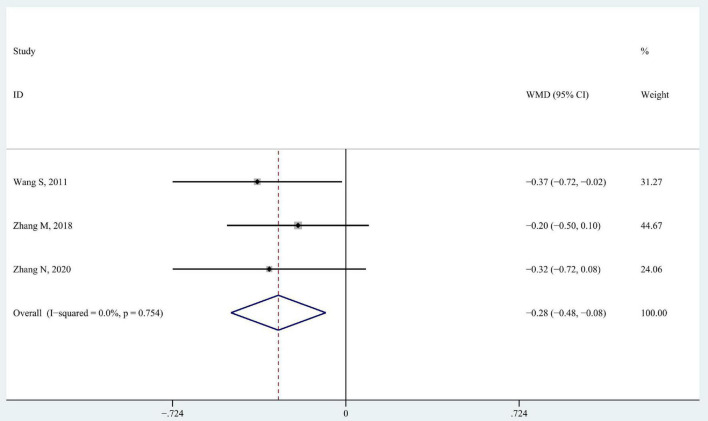
Forest plot of pooled results for modified Ashworth scale.

Modified Ashworth scale includes six levels to describe muscular tone (0, 1, 1+, 2, 3, 4, from normal to high). We replaced these six levels with scores ranging from 0 to 5 from low to high in data analysis. Three studies recruited children with spastic CP and used MAS to measure the changes in spasticity (Wang et al., 2011; Zhang and Liu, 2018; Zhang et al., 2020). The results showed that the MAS level decreased more in intervention groups than in control groups when data from the three studies were pooled together (WMD −0.28; 95% CI −0.48 to −0.08, *P* = 0.005, *I*^2^ = 0%) (Figure 5).

#### 3.3.3. Berg balance scale

**FIGURE 6:**
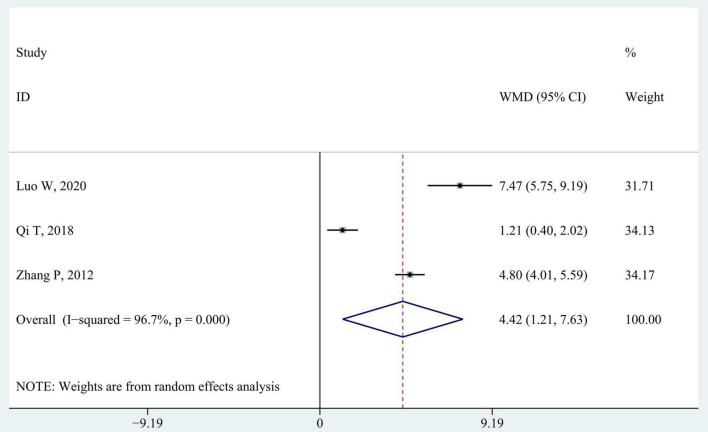
Forest plot of pooled results for Berg balance scale.

Berg balance scale is used in three studies to evaluate static and dynamic balance (Zhang and Hu, 2012; Qi and Wang, 2018; Luo et al., 2020). As shown in Figure 6, children in intervention groups achieved significantly better improvement in balance ability than those in control groups (WMD 4.42; 95% CI 1.21–7.63, *P* = 0.007, *I*^2^ = 96.7%). There was still significant heterogeneity between studies (all *I*^2^ > 80%) when the three studies were eliminated from the pooled data gradually through the sensitivity analysis.

#### 3.3.4. Activities of daily living

**FIGURE 7:**
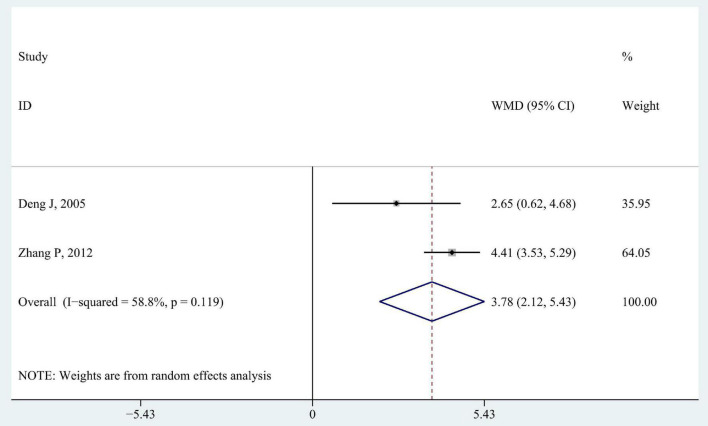
Forest plot of pooled results for activities of daily living.

Two studies reported ADL scores (Figure 7; Deng et al., 2005; Zhang and Hu, 2012). The pooled analysis reported a significantly better improvement in ADL scores in intervention groups than that in control groups (WMD 3.78; 95% CI 2.12–5.43, *P* < 0.001, *I*^2^ = 58.8%).

### 3.4. Quality appraisal

**FIGURE 8:**
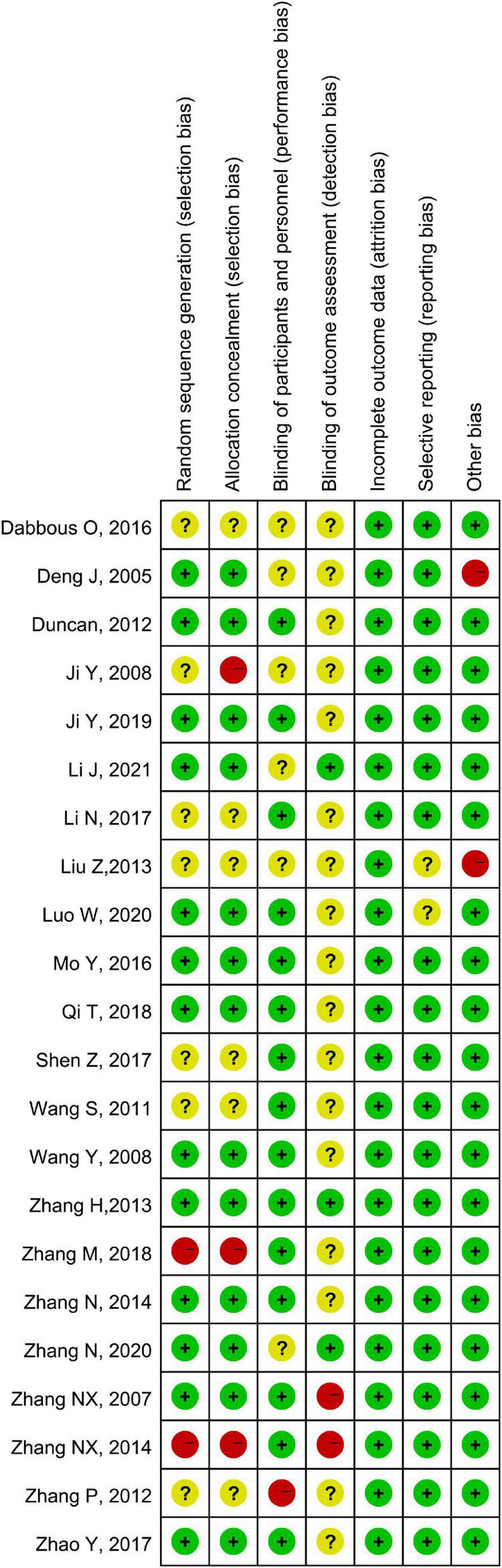
The bias chart of the included studies assessed by the Cochrane risk of bias tool.

According to the Cochrane risk of bias tool, seven studies had a high risk of bias, while 14 studies showed an unclear risk of bias, with only one study having a low risk of bias (Table 3 and Figure 8). The quality of evidence of the included outcome measures is shown in Table 4, with only one outcome measure (GMFM-88–Dimension A) showing high quality, while five of the thirteen outcome measures, including GMFM-88 Dimension B, C, D, and E, and MAS, have moderate quality.

**TABLE 3:** The Cochrane collaboration’s tool of assessing risk of bias for methodological assessment.

References	Random sequence generation	Allocation concealment	Blinding of participants and personnel	Blinding of outcome assessments	Incomplete outcome data	Selective reporting	Other bias	Overall bias
Dabbous et al., 2016	Unclear	Unclear	Unclear	Unclear	Low	Low	Low	Unclear
Deng et al., 2005	Low	Low	Unclear	Unclear	Low	Low	High	High
Duncan et al., 2012	Low	Low	Low	Unclear	Low	Low	Low	Unclear
Ji et al., 2008	Unclear	High	Unclear	Unclear	Low	Low	Low	High
Ji et al., 2019	Low	Low	Low	Unclear	Low	Low	Low	Unclear
Li J. et al., 2021	Low	Low	Unclear	Low	Low	Low	Low	Unclear
Li et al., 2017	Unclear	Unclear	Low	Unclear	Low	Low	Low	Unclear
Liu et al., 2013	Unclear	Unclear	Unclear	Unclear	Low	Unclear	High	High
Luo et al., 2020	Low	Low	Low	Unclear	Low	Unclear	Low	Unclear
Mo et al., 2016	Low	Low	Low	Unclear	Low	Low	Low	Unclear
Qi and Wang, 2018	Low	Low	Low	Unclear	Low	Low	Low	Unclear
Shen et al., 2017	Unclear	Unclear	Low	Unclear	Low	Low	Low	Unclear
Wang et al., 2011	Unclear	Unclear	Low	Unclear	Low	Low	Low	Unclear
Wang et al., 2008	Low	Low	Low	Unclear	Low	Low	Low	Unclear
Zhang and Du, 2013	Low	Low	Low	Low	Low	Low	Low	Low
Zhang and Liu, 2018	High	High	Low	Unclear	Low	Low	Low	High
Zhang N. et al., 2014	Low	Low	Low	Unclear	Low	Low	Low	Unclear
Zhang et al., 2020	Low	Low	Unclear	Low	Low	Low	Low	Unclear
Zhang et al., 2007	Low	Low	Low	High	Low	Low	Low	High
Zhang N. X. et al., 2014	High	High	Low	High	Low	Low	Low	High
Zhang and Hu, 2012	Unclear	Unclear	High	Unclear	Low	Low	Low	High
Zhao et al., 2017	Low	Low	Low	Unclear	Low	Low	Low	Unclear

**TABLE 4:** Quality of evidence measured by grading of recommendation assessment, development, and evaluation.

Patient or population: Children with cerebral palsy Settings: Intervention: Integrated Traditional Chinese medicine and modern rehabilitation therapies						
Outcomes	Illustrative comparative risks* (95% CI)		Relative effect (95% CI)	No of participants (studies)	Quality of the evidence (GRADE)	Comments
	Assumed risk	Corresponding risk				
	Control	Integrated traditional Chinese medicine and conventional rehabilitation therapies				
Gross motor function measure–66 questionnaire		The mean gross motor function measure–66 in the intervention groups was **9.33 higher** (0.14–18.52 higher)		285 (three studies)	⊕⊕⊝⊝ **Low**^1,2^	
Gross motor function measure–88 questionnaire		The mean gross motor function measure–88 in the intervention groups was **8.24 higher** (3.25–13.24 higher)		160 (two studies)	⊕⊕⊝⊝ **Low**^1,2^	
Gross motor function measure–88–Dimension A questionnaire		The mean gross motor function measure–88–Dimension A in the intervention groups was **6.63 higher** (5.36–7.91 higher)		580 (three studies)	⊕⊕⊕⊕ **High**	
Gross motor function measure–88–Dimension B questionnaire		The mean gross motor function measure–88–Dimension B in the intervention groups was **5.76 higher** (3.20–8.13 higher)		811 (five studies)	⊕⊕⊕⊝ **Moderate**^1^	
Gross motor function measure–88–Dimension C questionnaire		The mean gross motor function measure–88–Dimension C in the intervention groups was **4.83 higher** (2.62–7.04 higher)		691 (four studies)	⊕⊕⊕⊝ **Moderate**^1^	
Gross motor function measure–88–Dimension D questionnaire		The mean gross motor function measure–88–Dimension D in the intervention groups was **2.88 higher** (1.33–4.43 higher)		751 (five studies)	⊕⊕⊕⊝ **Moderate**^1^	
Gross motor function measure–88–Dimension E questionnaire		The mean gross motor function measure–88–Dimension E in the intervention groups was **4.49 higher** (0.82 lower to 9.79 higher)		691 (four studies)	⊕⊕⊕⊝ **Moderate**^1^	
Peabody Developmental Motor Scales–Grasping questionnaire		The mean Peabody Developmental Motor Scales–Grasping in the intervention groups was **3.46 higher** (1.77 lower to 8.70 higher)		238 (two studies)	⊕⊕⊝⊝ **Low**^1,2^	
Peabody Developmental Motor Scales–Visual motor integration questionnaire		The mean Peabody Developmental Motor Scales–Visual motor integration in the intervention groups was **3.08 higher** (2.78 lower to 8.93 higher)		238 (two studies)	⊕⊕⊝⊝ **Low**^1,2^	
Joint range of motion–Wrist extension protractor		The mean Joint range of motion– Wrist extension in the intervention groups was **4.13 higher** (0.79 lower to 9.04 higher)		158 (two studies)	⊕⊕⊝⊝ **Low**^1,2^	
Modified Ashworth scale (MAS) questionnaire		The mean Modified Ashworth scale (MAS) in the intervention groups was **0.28 lower** (0.48 to 0.08 lower)		325 (three studies)	⊕⊕⊕⊝ **Moderate**^2^	
Berg balance scale (BBS) questionnaire		The mean Berg balance scale (BBS) in the intervention groups was **4.42 higher** (1.21 to 7.63 higher)		231 (three studies)	⊕⊕⊝⊝ **Low**^1,2^	
Activities of daily living (ADL) questionnaire		The mean activities of daily living (ADL) in the intervention groups was **3.78 higher** (2.12–5.43 higher)		150 (two studies)	⊕⊕⊝⊝ **Low**^1,2^	

The basis for the assumed risk (e.g., the median control group risk across studies) is provided in footnotes. The corresponding risk (and its 95% confidence interval) is based on the assumed risk in the comparison group and the relative effect of the intervention (and its 95% CI). CI, confidence interval. GRADE Working Group grades of evidence. High quality: Further research is very unlikely to change our confidence in the estimate of effect. Moderate quality: Further research is likely to have an important impact on our confidence in the estimate of effect and may change the estimate. Low quality: Further research is very likely to have an important impact on our confidence in the estimate of effect and is likely to change the estimate. Very low quality: We are very uncertain about the estimate. ^1^*I*^2^ > 50% ^2^There were less than 400 participants in total.

## 4. Discussion

Our study found that integrated TCM and modern rehabilitation therapies significantly improved motor development in children with CP (ΔGMFM-66 score: 9.33, ΔGMFM-88 score: 8.24, ΔBerg balance scale score: 4.42), reduced muscle tone (ΔMAS score: −0.28), and increased the functional independence (ΔADL score: 3.78). These results suggested that TCM treatments combined with modern rehabilitation therapies may be an effective package of intervention for children with CP, compared to modern rehabilitation therapies only.

Developmental delay, especially motor development, is a symptom of widespread concern for children with CP. The brain remodeling theory is the basis of modern rehabilitation to improve motor development in children with CP (Aisen et al., 2011). Brain remodeling refers to the plasticity and modifiability of the brain. The neurons in the brain may reconnect by external stimuli, thus compensating for the dysfunction caused by brain damage (Gulyaeva, 2017). Modern rehabilitation therapy may help children with CP *via* neuroplasticity that reshapes the brain and compensates for the development delay (Dancause and Nudo, 2011). For example, the contralateral movement evoked field and ipsilateral motor field of the cortex were activated and reorganization in children with CP after constraint-induced movement therapy (Sutcliffe et al., 2007). Different from the brain remodeling theory of modern rehabilitation therapy, the mechanism of TCM improving motor development may be the blood flow regulation for the brain, which might promote the development of neurons and synaptic interconnection (Shepherd et al., 2018; Niu et al., 2019). Especially in scalp acupuncture, some special acupoints, such as DU-20 and X-HN1, may expand the blood vessels of the corresponding brain areas or promote collateral circulation (Chinese Association of Rehabilitation Medicine Pediatric Rehabilitation Committee et al., 2022b). Expression of endothelin receptor type A, which was associated with vasoconstriction, decreased in mice after being treated with acupuncture (Li J. et al., 2021). Some studies demonstrated that another mechanism of TCM in improving motor development might be the mediation of neurotransmitters, such as gamma-aminobutyric acid, an inhibitory neurotransmitter for motoneurons to improve motor development (Shorter and Segesser, 2013).

Our systematic review showed that the spasticity was improved by the integrated TCM and modern rehabilitation therapies, compared to modern rehabilitation therapy only. In the theory of TCM, children with spastic CP may have a constitution with Yin-deficiency (Zhang et al., 2016). The kidney stores the essence of life and is responsible for body development. The liver is the organ that stores blood. The spleen-stomach is responsible for digestion and nutrient absorption, which is then converted into Qi-blood-body fluid for brain and body development. Therefore, the treatment for spastic CP should aim at brain recovery and start with the Yin deficiency. Acupuncture, massage, or herbal fumigation should consider tonifying Yin deficiency *via* the acupoints on Yin meridians (such as spleen, liver, and kidney meridians) based on the dialectical diagnosis. With regard to the mechanism related to the effect of the TCM treatments on spasticity, one study suggested that scalp acupuncture may increase and disentangle the white matter fiber bundles in rats with CP (Wang et al., 2021), which may be the anatomical evidence for improving spasticity. A neuroimaging study showed that acupuncture on LR3 may relieve the spasticity of children with CP by reducing the activation of the frontal lobe cortex, an important brain region that controls muscle tone and active movement (Wu et al., 2008).

However, no significant improvement was found in fine motor development in the integrated TCM and modern rehabilitation therapies group, compared to the control group. The possible explanation might be that fine motor improvement requires targeted training. Evidence supported that task-oriented motor training based on the requirements of daily routines may be effective in the improvement of fine motor development (Baker et al., 2022). With regard to unilateral hand function, constraint-induced movement therapy may be effective in children who were diagnosed with unilateral CP (Tinderholt Myrhaug et al., 2014). However, few included studies in our systematic review employed targeted training for fine motor in the conservative rehabilitation protocol. Massage and acupuncture were also recommended for fine motor development delay in children with CP, but individualized treatment should be considered because children with CP vary in the syndrome classifications based on the TCM theory (Chinese Association of Rehabilitation Medicine Pediatric Rehabilitation Committee et al., 2022b). Our results suggest that targeted and individualized therapy should be added to promote fine motor development in children with CP.

Seventeen studies declared that none of the participants withdrew from the studies and no severe adverse event was reported in any of the enrolled studies, indicating that TCM treatments are safe in the clinical setting for children with CP. The duration of intervention for children with CP ranged from 20 days to 6 months with a median intervention time of 3 months. Future studies should focus on the long-term effects of TCM treatments on CP.

### 4.1. Limitations

There were some limitations of our systematic review, which should be interpreted with caution. First, methodological heterogeneity in the included should not be ignored. Due to the differences in the age and CP severity of the participants, TCM treatments varied among the included studies, such as acupuncture, massage, and herbal fumigation. Subgroup analysis of different intervention protocols could not be conducted because of the insufficient number of included studies. Future studies should consider standardized TCM diagnosis and treatment for children with CP. Second, the pooled results of our systematic review may suffer from methodological quality. Seven of the included studies showed a high risk of bias, while only two studies specified the blinding of the assessors. Third, 21 of the 22 included studies are from China, which indicates that the integrated TCM and modern rehabilitation therapies for CP are not widely used in the world. In the future, the promotion of TCM needs to be strengthened, such as the training of international TCM practitioners. Fourth, it is difficult to quantitatively divide the proportion of TCM treatments and modern rehabilitation therapies because of the differences in basic theory between TCM and modern rehabilitation.

### 4.2. Implications for clinical practice and research

This systematic review with meta-analysis suggests that TCM may be integrated into the traditional rehabilitation treatment of children with cerebral palsy to improve gross motor development and regulate muscle tone. With regard to fine motor improvement, further studies on the targeted and individualized treatment protocol for children with CP should be noticed. For example, meridians and acupoints should be selected based on syndrome differentiation.

Children with CP also showed a significant increase in functional independence after the treatment of integrated TCM and modern rehabilitation therapies compared to modern rehabilitation therapies only. More studies will be needed in the future to explore the long-term effect of integrated therapies on activities of daily living, given that long-term application of the integrated therapies to children with CP may help them to return to school in adolescence and return to society in adulthood.

Traditional Chinese medicine therapies included in this systematic review consist of body and scalp acupuncture, massage, and fumigation. The methodological heterogeneity needs attention in this systematic review, which may be due to the diversity of TCM treatments. Future studies should focus on the standardization of TCM diagnosis and treatment for children with CP. To promote the application of TCM worldwide, an international training program of TCM should be established to increase the accreditation of TCM practitioners.

## 5. Conclusion

This systematic review indicated that integrated TCM and modern rehabilitation therapies may be recognized as an effective and safe therapy to improve gross motor function, reduce muscle tone, and improve the functional independence of children with CP, compared to modern rehabilitation therapy only. Due to the methodological heterogeneity and the potential risk of bias in the included studies, our results should be interpreted with caution. Future studies should focus on the standardization of TCM treatments, and training of international TCM practitioners may be considered for TCM promotions.

## Data availability statement

The raw data supporting the conclusions of this article will be made available by the authors, without undue reservation.

## Author contributions

ZC and ZH contributed to the studies retrieval and data extraction. QD and XZ designed and developed the framework for the manuscript. All authors contributed to the article’s writing, read the manuscript, and agreed to submit this version.
