# Supplementary material for: Effects of traditional Chinese medicine combined with modern rehabilitation therapies on motor function in children with cerebral palsy: A systematic review and meta-analysis
Source: Front Neurosci. 2023 Feb 8;17:1097477. doi: 10.3389/fnins.2023.1097477 (PMC9944433; doi:10.3389/fnins.2023.1097477)
Supplement: Supplementary file 1 [file Data_Sheet_1.PDF]

PubMed

#1 “Single-Blind Method”[MeSH Terms] OR "Double-Blind Method"[MeSH Terms] OR "Randomized Controlled Trials As Topic"[MeSH Terms] OR "Randomized Controlled Trial" [Publication Type] OR "Intention To Treat Analysis"[MeSH Terms] OR "Controlled Clinical Trials As Topic"[MeSH Terms] OR "Clinical Trials As Topic"[MeSH Terms] OR "Clinical Trial" [Publication Type] OR Randomized Controlled Trial[Publication Type]

#2 "Random*"[Text Word] OR Allocation[Text Word] OR "Random Allocation"[Text Word] OR Placebo[Text Word] OR Single Blind[Text Word] OR Double Blind[Text Word] OR "Randomized Controlled Trial*"[Text Word] OR RCT[Text Word]

#3 #1 OR #2

#4 Animals NOT Humans

#5 #3 NOT #4

#6 “Cerebral palsy”[MeSH Terms] OR “Cerebral palsy”[Title/Abstract] OR “Cerebral paralysis”[Title/Abstract] OR “Spastic quadriplegia”[Title/Abstract] OR “Spastic diplegia”[Title/Abstract] OR “Spastic hemiplegia”[Title/Abstract] OR “Little disease”[Title/Abstract]

#7 “Medicine, Chinese traditional”[MeSH Terms] OR “Chinese medicine”[Title/Abstract] OR “Traditional medicine”[Title/Abstract] OR “Acupuncture therapy”[MeSH Terms] OR “Acupuncture”[Title/Abstract] OR “Acupotomy”[Title/Abstract] OR “Acupotomology”[Title/Abstract] OR “Acupotome”[Title/Abstract] OR “needle”[Title/Abstract] OR “needling”[Title/Abstract] OR “moxibustion”[Title/Abstract] OR “Tuina”[Title/Abstract] OR “Chinese massage”[Title/Abstract] OR “Cupping”[Title/Abstract] OR “Manipulation”[Title/Abstract] OR “Drugs, Chinese herbal”[MeSH Terms] OR “herb*”[Title/Abstract] OR “collateral channels”[Title/Abstract] OR “Qigong”[MeSH Terms] OR “Tai chi”[Title/Abstract] OR “Tai ji”[Title/Abstract] OR “Tai-chi”[Title/Abstract] OR “baduanjin”[Title/Abstract]

#8 “motor skills”[MeSH Terms] OR “motor”[Title/Abstract] OR “motor development”[Title/Abstract] OR “gross motor”[Title/Abstract] OR “fine motor”[Title/Abstract] OR “movement”[Title/Abstract] OR “postur*” OR “walk*”[Title/Abstract] OR “sit”[Title/Abstract] OR “sitting”[Title/Abstract] OR “crawl*”[Title/Abstract] OR “gait”[MeSH Terms] OR “walking speed”[Title/Abstract] OR “balance”[Title/Abstract] OR “range of motion”[Title/Abstract] OR “flexibility”[Title/Abstract] OR “passive motion”[Title/Abstract] OR “Gross Motor Function Measure”[Title/Abstract] OR “GMFM”[Title/Abstract] OR “modified ashworth scale”[Title/Abstract] OR “peabody”[Title/Abstract]

#9 "Infant"[MeSH Terms] OR "Child"[MeSH Terms] OR "Adolescent"[MeSH Terms] OR "Pediatrics"[MeSH Terms] OR Infant[Title/Abstract] OR Preschool[Title/Abstract] OR Child[Title/Abstract] OR Children[Title/Abstract] OR Adolescent[Title/Abstract] OR Adolescents[Title/Abstract] OR Adolescence[Title/Abstract] OR Teens[Title/Abstract] OR Teen[Title/Abstract] OR Teenagers[Title/Abstract] OR Teenager[Title/Abstract] OR Youth[Title/Abstract] OR Youths[Title/Abstract] OR Girl[Title/Abstract] OR Girls[Title/Abstract] OR Boy[Title/Abstract] OR Boys[Title/Abstract] OR Pediatric[Title/Abstract] OR Pediatrics[Title/Abstract] OR Paediatric[Title/Abstract] OR

Paediatrics[Title/Abstract]
#10 #5 AND #6 AND #7 AND #8 AND #9

Cochrane Library
#1 parallel:ti,ab,kw OR observational:ti,ab,kw OR cross-sectional:ti,ab,kw OR pre-post:ti,ab,kw OR before-after:ti,ab,kw OR 'controlled trial*':ti,ab,kw OR 'random*':ti,ab,kw OR 'randomi*':ti,ab,kw OR 'intervention*':ti,ab,kw
#2 trial:pt
#3 #1 OR #2
#4 MeSH descriptor: [cerebral palsy] explode all trees
#5 ‘Cerebral palsy’:ti,ab,kw OR 'Spastic quadriplegia':ti,ab,kw OR 'Spastic diplegia':ti,ab,kw OR 'Spastic hemiplegia':ti,ab,kw OR 'Little disease':ti,ab,kw
#6 #4 OR #5
#7 MeSH descriptor: [Medicine, Chinese Traditional]explode all trees
#8 MeSH descriptor: [Acupuncture]explode all trees
#9 'Chinese medicine':ti,ab,kw OR 'Traditional medicine':ti,ab,kw OR 'Acupuncture':ti,ab,kw OR 'Acupotomy':ti,ab,kw OR 'Acupotomology':ti,ab,kw OR 'Acupotome':ti,ab,kw OR 'Needle':ti,ab,kw OR 'Needling':ti,ab,kw OR 'Moxibustion ':ti,ab,kw OR 'Tuina':ti,ab,kw OR 'Chinese massage':ti,ab,kw OR 'Cupping':ti,ab,kw OR 'Manipulation':ti,ab,kw OR 'herb*':ti,ab,kw OR 'collateral channels':ti,ab,kw OR 'Qigong':ti,ab,kw OR 'Tai chi':ti,ab,kw OR 'Tai ji':ti,ab,kw OR 'Tai-chi':ti,ab,kw OR 'baduanjin':ti,ab,kw
#10 #7 OR #8 OR #9
#11 MeSH descriptor: [gait]explode all trees
#12 MeSH descriptor: [postural balance]explode all trees
#13 'motor skills':ti,ab,kw OR 'motor':ti,ab,kw OR 'motor development':ti,ab,kw OR 'gross motor':ti,ab,kw OR 'fine motor':ti,ab,kw OR 'movement':ti,ab,kw OR 'postur*':ti,ab,kw OR 'walk*':ti,ab,kw OR 'sit':ti,ab,kw OR ‘sitting’:ti,ab,kw OR 'crawl*':ti,ab,kw OR 'gait':ti,ab,kw OR 'walking speed':ti,ab,kw OR 'balance':ti,ab,kw OR ‘range of motion':ti,ab,kw OR ‘flexibility':ti,ab,kw OR 'passive motion':ti,ab,kw OR 'Gross Motor Function Measure':ti,ab,kw OR 'GMFM':ti,ab,kw OR 'modified ashworth scale':ti,ab,kw OR 'peabody':ti,ab,kw
#14 #11 OR #12 OR #13
#15 MeSH descriptor: [Infant] explode all trees
#16 MeSH descriptor: [Child] explode all trees
#17 MeSH descriptor: [Adolescent] explode all trees
#18 MeSH descriptor: [Pediatrics] explode all trees
#19 Infant:ti,ab,kw OR Preschool:ti,ab,kw OR Child:ti,ab,kw OR Children:ti,ab,kw OR Adolescent:ti,ab,kw OR Adolescents:ti,ab,kw OR Adolescence:ti,ab,kw OR Teens:ti,ab,kw OR Teen:ti,ab,kw OR Teenagers:ti,ab,kw OR Teenager:ti,ab,kw OR Youth:ti,ab,kw OR Youths:ti,ab,kw OR Girl:ti,ab,kw OR Girls:ti,ab,kw OR Boy:ti,ab,kw OR Boys:ti,ab,kw OR Pediatric:ti,ab,kw OR Pediatrics:ti,ab,kw OR Paediatric:ti,ab,kw OR

Paediatrics:ti,ab,kw
#20 #15 OR #16 OR #17 OR #18 OR #19
#21 #3 AND #6 AND #10 AND #14 AND #20

EMBASE

#1 'parallel'/exp OR 'observational'/exp OR 'cross-sectional'/exp OR 'pre-post'/exp OR 'before-after'/exp OR 'controlled trial*'/exp OR 'random*'/exp OR ' randomi*'/exp OR 'intervention*'/exp

#2 random*:ab,ti OR observational:ab,ti OR cohor:ab,ti OR cross-sectional:ab,ti OR parallel:ab,ti OR pre-post:ab,ti OR before-after:ab,ti

#3 #1 OR #2

#4 'Cerebral palsy'/exp OR 'Cerebral palsy':ab,ti OR 'Spastic quadriplegia':ab,ti OR 'Spastic diplegia':ab,ti OR 'Spastic hemiplegia':ab,ti OR 'Little disease':ab,ti

#5 'Chinese medicine'/exp OR 'Chinese medicine':ab,ti OR 'Traditional Chinese medicine':ab,ti OR 'Acupuncture therapy'/exp OR 'Acupotomy':ab,ti OR 'Acupotomology':ab,ti OR 'Acupotome':ab,ti OR 'Needle':ab,ti OR 'Needling':ab,ti OR 'Moxibustion ':ab,ti OR 'Tuina':ab,ti OR 'Chinese massage':ab,ti OR 'Cupping':ab,ti OR 'Manipulation':ab,ti OR 'herb*':ab,ti OR 'collateral channels':ab,ti OR 'Qigong'/exp OR 'Tai chi':ab,ti OR 'Tai ji':ab,ti OR 'Tai-chi':ab,ti OR 'baduanjin':ab,ti OR 'baduanjin exercise':ab,ti

#6 'gross motor':ab,ti OR 'fine motor':ab,ti OR 'movement':ab,ti OR 'postur*' OR 'sit':ab,ti OR 'sitting':ab,ti OR 'crawl*':ab,ti OR 'gait'/exp OR 'walking speed':ab,ti OR 'balance':ab,ti OR 'range of motion':ab,ti OR ‘flexibility':ab,ti OR 'passive motion':ab,ti OR 'Gross Motor Function Measure':ab,ti OR 'GMFM':ab,ti OR 'modified ashworth scale':ab,ti OR 'peabody':ab,ti OR 'motor':ab,ti OR 'motor development':ab,ti

#7 'Child'/exp OR 'Adolescent'/exp OR 'Pediatrics'/exp OR Infant:ab,ti OR Preschool:ab,ti OR Child:ab,ti OR Children:ab,ti OR Adolescent:ab,ti OR Adolescents:ab,ti OR Adolescence:ab,ti OR Teens:ab,ti OR Teen:ab,ti OR Teenagers:ab,ti OR Teenager:ab,ti OR Youth:ab,ti OR Youths:ab,ti OR Girl:ab,ti OR Girls:ab,ti OR Boy:ab,ti OR Boys:ab,ti OR Pediatric:ab,ti OR Pediatrics:ab,ti OR Paediatric:ab,ti OR Paediatrics:ab,ti

#8 #3 AND #4 AND #5 AND #6 AND #7

WOS

#1 TS=(parallel OR observational OR cross-sectional OR pre-post OR before-after OR controlled trial* OR random* OR randomi* OR intervention*)

#2 TS=('Cerebral palsy' OR 'Spastic quadriplegia' OR 'Spastic diplegia' OR 'Spastic hemiplegia' OR 'Little disease')

#3 TS=('Chinese medicine' OR 'Traditional medicine' OR 'Acupuncture’ OR 'Acupotomy' OR 'Acupotomology' OR 'Acupotome' OR 'Needle' OR 'Needling' OR 'Moxibustion' OR 'Tuina' OR 'Chinese massage' OR 'Cupping' OR 'Manipulation' OR 'herb*' OR 'collateral channels' OR 'Qigong' OR 'Tai chi' OR 'Tai ji' OR 'Tai-chi' OR 'baduanjin')

#4 TS=('motor skills' OR 'motor development' OR 'gross motor' OR 'fine motor' OR 'movement' OR 'postur*' OR 'walk*' OR 'sit*' OR 'crawl*' OR 'gait' OR 'walking speed' OR 'balance' OR ‘range of motion' OR ‘flexibility' OR 'passive motion' OR 'Gross Motor Function Measure' OR 'GMFM' OR 'modified ashworth scale' OR 'peabody')

#5 TS=(Infant OR Preschool OR Child OR Children OR Adolescent OR Adolescents OR Adolescence OR Teens OR Teen OR Teenagers OR Teenager OR Youth OR Youths OR Girl OR Girls OR Boy OR Boys OR Pediatric OR Pediatrics OR Paediatric OR Paediatrics)

#6 #1 AND #2 AND #3 AND #4 AND #5

CINAHL

S1 MH("Random Assignment" OR "Placebos" OR "Placebo Effect" OR "Single-Blind Studies" OR "Double-Blind Studies" OR "Triple-Blind Studies" OR "Randomized Controlled Trials" OR "comparative studies" OR "Evaluation Research" OR "Prospective Studies" OR "crossover Design" OR "Prospective Studies" OR "Clinical Trials" OR "Clinical Trial Registry")

S2 TX (random$ OR allocation OR "random allocation" OR placebo$ OR single blind OR double blind OR "randomi?ed controlled trial*" OR "controlled clinical trial*" OR "comparative study" OR "evaluation stud*" OR "follow-up stud*" OR "prospective stud*" OR "cross-over stud*" OR control$ OR prospectiv$ OR volunteer$ OR "RCT" OR "clinical trial*")

S3 PT (randomized controlled trial OR "clinical trial*")

S4 S1 OR S2 OR S3

S5 MH("Cerebral palsy+") OR AB ("Cerebral palsy" OR "Spastic quadriplegia" OR "Spastic diplegia" OR "Spastic hemiplegia" OR "Little disease")

S6 MH ("Medicine, Chinese Traditional+") OR AB ("Chinese medicine" OR "Traditional medicine" OR "Acupuncture" OR "Acupotomy" OR "Acupotomology" OR "Acupotome" OR "Needle" OR "Needling" OR "Moxibustion " OR "Tuina" OR "Chinese massage" OR "Cupping" OR "Manipulation" OR "herb*" OR "collateral channels" OR "Qigong" OR "Tai chi" OR "Tai ji" OR "Tai-chi" OR "baduanjin")

S7 AB ("gross motor" OR "fine motor" OR "movement" OR "postur*" OR "sit" OR “sitting” OR "crawl*" OR "gait" OR "walking speed" OR "balance" OR " range of motion" OR " flexibility" OR "passive motion" OR "Gross Motor Function Measure" OR "GMFM" OR "modified ashworth scale" OR "peabody" OR "motor" OR "motor development")

S8 MH("Child+" OR "Adolescence+" OR "Pediatrics+") OR AB(Infant OR Preschool OR Child OR Children OR Adolescent OR Adolescents OR Adolescence OR Teens OR Teen OR Teenagers OR Teenager OR Youth OR Youths OR Girl OR Girls OR Boy OR Boys OR Pediatric OR Pediatrics OR Paediatric OR Paediatrics)

S9 S4 AND S5 AND S6 AND S7 AND S8
